# Viewpoint: Virtual and Augmented Reality in Basic and Advanced Life Support Training

**DOI:** 10.2196/28595

**Published:** 2022-03-23

**Authors:** Serena Ricci, Andrea Calandrino, Giacomo Borgonovo, Marco Chirico, Maura Casadio

**Affiliations:** 1 Department of Informatics, Bioengineering, Robotics, and Systems Engineering University of Genova Genova Italy; 2 Simulation and Advanced Education Center University of Genova Genova Italy; 3 Pediatric Emergency and Critical Care Department Giannina Gaslini children’s Hospital Genova Italy; 4 Department of Surgical Sciences and Integrated Diagnostics University of Genova Genova Italy

**Keywords:** basic and advanced life support, first aid, cardiopulmonary resuscitation, emergency, training, simulation training, medical simulation, healthcare simulation, virtual reality, augmented reality

## Abstract

The use of augmented reality (AR) and virtual reality (VR) for life support training is increasing. These technologies provide an immersive experience that supports learning in a safe and controlled environment. This review focuses on the use of AR and VR for emergency care training for health care providers, medical students, and nonprofessionals. In particular, we analyzed (1) serious games, nonimmersive games, both single-player and multiplayer; (2) VR tools ranging from semi-immersive to immersive virtual and mixed reality; and (3) AR applications. All the toolkits have been investigated in terms of application goals (training, assessment, or both), simulated procedures, and skills. The main goal of this work is to summarize and organize the findings of studies coming from multiple research areas in order to make them accessible to all the professionals involved in medical simulation. The analysis of the state-of-the-art technologies reveals that tools and studies related to the multiplayer experience, haptic feedback, and evaluation of user’s manual skills in the foregoing health care-related environments are still limited and require further investigation. Also, there is an additional need to conduct studies aimed at assessing whether AR/VR-based systems are superior or, at the minimum, comparable to traditional training methods.

## Introduction

Life support training has become more important in recent decades, creating mass training possibilities that have the potential to significantly reduce the number of deaths due to sudden cardiac arrest [1,2]. In emergency health care, it is crucial to know the differences between advanced training targeted at qualified professionals and training of the general population and paramedics [3]. Qualified professionals need to know how to use mechanical tools and drugs, in addition to performing lifesaving tasks, and general population cohorts need to know and understand how to perform basic manual skills (eg, maintaining an open airway and performing chest compressions) while waiting for professional help. It is important that both professionals and untrained rescuers are able to train with safe and realistic emergency medical scenarios. Training should provide opportunities for frequent rehearsal and assessment of a required core knowledge base in order to achieve optimal levels of practical expertise in stressful situations [4]. Life support training is typically accomplished via the use of manikins and simulated scenarios; moreover, the use of disruptive technologies like virtual reality (VR) and augmented reality (AR) have garnered more interest in training [5-7], beginning with nonimmersive serious games, up to immersive VR, AR, and mixed reality (MR) that provides haptic feedback and realistic interactions. This is probably due to the fact that these technologies can enhance immersivity, defined as the subjective impression to be part of a realistic experience [8], which further strengthens medical learning [9-11]. That said, efficacy studies on VR and AR simulations for emergency training have not been sufficiently performed, as the majority of the research studies in the area are proofs-of-concept. In fact, studies in the field have been presented in scientific journals and conferences that include many different research areas, such as computer science, engineering, medicine, and simulation. The resulting fragmentation and mix-and-match of studies in immersive medical emergency training have resulted in a jumble of different terminologies and aims, making it difficult to have a comprehensive overview of many existing immersive applications to date.

Our goal for this paper is to provide a comprehensive analysis and review of state-of-the-art of VR- and AR-based simulators for life support training. Our intention is to target professionals involved in medical simulation (ie, physicians, medical instructors, simulation specialists, engineers, technicians, computer scientists, and VR and AR developers).

We include, in our review, studies from multiple research areas, such as medicine, engineering, and computer science (Multimedia Appendix 1). First, we provide a description of VR- and AR-based tools used for first aid training, classifying them according to their technology, main features, and appropriate end-users; we also emphasize the advantages and limitations of the foregoing technologies. Following this, we pursue a discussion of the primary properties a simulator should have for optimizing trainee and instructor educational needs.

As the different studies cited are not of the same quality—some of them are well-designed randomized controlled trials, whereas others are prepost studies with surrogate outcomes and few subjects—we classified the studies according to the model of Kirkpatrick for the evaluation of outcomes [12], as this model is commonly used in the field of medical education [13]. Briefly, education outcomes can be classified in different levels as follows:

- Level 1: reaction to learning experience.
- Level 2a: modification of attitudes and perceptions.
- Level 2b: acquisition of knowledge and skills.
- Level 2c: retention of knowledge and skills over a period of time.
- Level 3: behavioral change.
- Level 4a: change in organizational practice.
- Level 4b: benefits to patients/clients, families, and communities.

**Figure 1:**
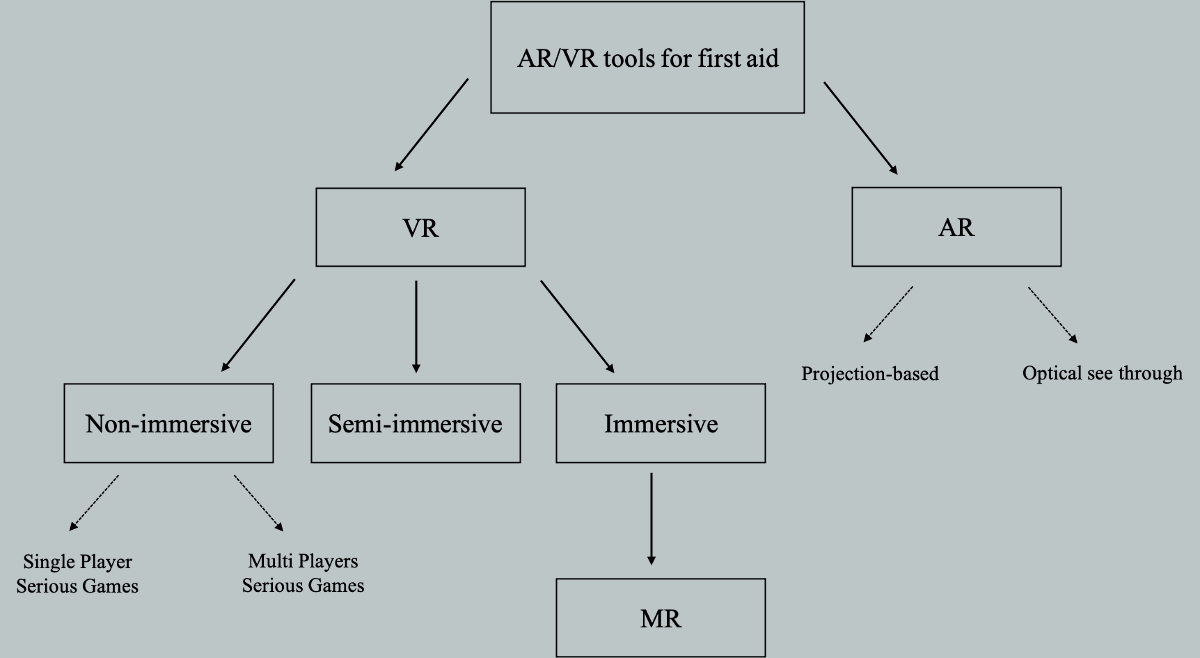
Organization of the review. AR: augmented reality; MR: mixed reality; VR: virtual reality.

The review is organized as shown in Figure 1. We provide an overview of VR, AR, and MR; then, the first part encompasses the primary research studies for VR for life support training. The second part of our review covers AR applications for emergency care training and assessment.

### Overview of VR and AR Technology

VR can be divided into nonimmersive, semi-immersive, and immersive VR (Textbox 1) [14,15].

**Definitions of virtual reality and augmented technology**

**Virtual reality: computer-generated simulation:**

- Nonimmersive: the virtual environment is accessed through a display, and interaction is limited to keyboard and mouse
- Semi-immersive: the setup includes projections or multiple screens
- Immersive: a head-mounted display isolates the user from the real world
  Mixed reality: virtual and real worlds are overlapped such that the user sees the virtual world and interacts with the real one

**Augmented reality: integration of 3D virtual objects into a real environment in real time:**

- Optical see through: semi-transparent glasses to combine the virtual content with the real view of the world
- Video see-through: cameras to show the user the real world, augmented with virtual elements
- Projection-based: the virtual elements are projected in the real world

Nonimmersive VR includes desktop applications, in which the virtual environment is accessed through a screen, and interaction is usually limited to the keyboard and mouse, but it can also involve other devices such as gaming controllers, joysticks, or graphic tablets. Semi-immersive VR allows the user to experience a 3D environment through projections or multiple screens, while immersive VR requires a wired or wireless head-mounted display (HMD) that can fully isolate the user from the real environment [16]. In VR, interactions can occur in multiple ways: by using controllers, motion tracking devices, or data gloves which also provide haptic feedback such as vibrations. In addition, supplementary devices such as Leap Motion (Ultraleap) or external cameras track the users’ movements, thus allowing them to naturally interact with the VR without wearing or holding external devices.

Recently, immersive VR has further evolved into mixed reality (MR), combining virtual and real information, such that the virtual and real worlds can interact [17]. In particular, MR allows the users to be immersed in a virtual world and to interact with the virtual objects as they would normally do in the real world (eg, by grasping or handling real objects) [18,19], given that the virtual and real worlds are overlapped.

Another approach to “augment” the environment information is by using AR. In particular, AR can be defined as the integration of 3D virtual objects into a real environment in real time [20-23]. AR can be divided into (1) optical see through using semi-transparent glasses to combine the virtual content with the real view of the world; (2) video seen through taking advantage of cameras to show the user the real world, augmented with virtual elements; (3) projection-based which does not need any wearable device, as the virtual elements are projected (Textbox 1).

The main difference between AR and MR is the visual feedback provided: the former combines real and virtual elements in the user’s field of view; the latter shows a completely virtual world. In addition, with AR, the interaction with virtual objects is limited to simple gestures; conversely, MR guarantees a realistic interaction with virtual objects as the virtual and real world are overlapped.

### Virtual Reality

The studies are organized into nonimmersive, semi-immersive, and immersive VR categories (Figure 1). For the sake of clarity, nonimmersive systems are defined as serious games throughout the text, as this term is most commonly used in health care simulation for the applications under study. Further, the immersive VR section contains a part about MR systems with haptic feedback, commonly referred to as hybrid tools.

#### Nonimmersive Systems or Serious Games

The early definitions of serious games described them as games with an educational purpose not primarily intended for entertainment [24]. Conversely, [25] suggests that the educational purpose of these games was most commonly deployed in a way that made them entertaining. More recently, the term serious game has been associated with videogames that include scoring systems that challenge users [26]. In particular, serious games are educational computer applications that teach a specific skill that can be transferred into real life [26,27]. With serious games, students can deal with a challenging situation by learning from mistakes [28,29]. They also find different strategies and can be rewarded by a score and through the possibility of exploring levels having increasing difficulty [27]. In 2021, Bedwell et al [30] defined the characteristics a serious game should have that can be summarized as such: rules and a specific challenging goal, a story told during the game, actions that the user can control, communication between the player and the game characters, and a scoring system.

Within the foregoing context, medical education is particularly suited for the development of serious games ranging from 2D applications to teach anatomy to 3D applications that train triage or surgery [31]. For the purpose of this review, we will discuss only VR-based serious games, specifically designed for life support training, with a specific focus on the difference between single-player and multiplayer tools (Table 1).

**Table 1:** Serious games specifically designed for emergency training.

Study	Topic	Target	Players	Features	Study design (N)	Kirkpatrick's level
Youngblood et al [32], 2008	CRM^a^	HCP^b^	4	Users can communicate vocally in real time and can perform clinical actions only if they are properly positioned with respect to the patient who responds to treatments and actions	Prepost (30)	2b
Creutzfeldt et al [33], 2012 *CPR-MVW*	CPR^c^	HP, NCP^d^	3	Trainees are avatars in the virtual world who need to cooperate to perform BLS	Case control (30)	2b
Buttussi et al [34], 2013 *EMSAVE*	ALS^e^	HCP	1	User plays the leader of an ALS team. When a correct task is selected, its execution and effects are shown. Otherwise, the game provides hints for self-correction. Debriefing at the end of the simulation	Prepost (40)	2c
Ribeiro et al [35], 2014 *SeGTE*	CPR	NP	1	Training and evaluation modes; debriefing	Prepost (31)	2b
Vankipuram et al [36], 2014	ALS	HCP	6	Each role receives specific feedback and can perform certain actions. The player responsible for compression interacts with the system using a haptic joystick that mimics the patient’s chest	Usability (96)	2a
Boada et al [37], 2015 *LISSA*	CPR	HCP, NP	1	The player has to save the victim applying CPR. Scoring system with penalties for wrong actions and delays. Two types of users: teacher and learner	Randomized trial (109)	2b
Drummond et al [38], 2017 *Staying Alive*	CPR	HCP, NP	1	The player learns the appropriate tasks to manage SCA. Actions are guided throughout the game	Randomized trial (79)	1
Latif et al [39], 2017 *LA-VIE*	CPR	NP	1	Serious game usable to test the CPR knowledge of nonprofessional laypersons. The user is presented with different situations and needs to perform the correct action.	Prepost (52)	2b
Gerard et al [40], 2018 *PediatricSim*	PALS^f^	HCP	1	User plays the role of a code leader, selecting assessments and treatments performed by avatars. Two modes: tutorial, which provides real-time feedback; nontutorial with feedback at the end of the simulation	Prepost (60)	2a
Aksoy et al [41], 2019 *3DMedSim tablet-based BLS *	CPR	HCP	1	Usable with a tablet. Two modes: training which guides the trainees during the emergency and self-test	Randomized trial (40)	2b

^a^CRM: crisis resource management.

^b^HCP: health care providers.

^c^CPR: cardiopulmonary resuscitation.

^d^NP: nonprofessionals.

^e^ALS: advanced life support.

^f^PALS: pediatric advanced life support.

#### Single Player

Single player is the most common type of serious games used for life support training. Typically, a trainer assesses a first aid scenario and decides which treatments the rescue team needs to perform. Serious games are mainly used by medical students, paramedics, and medical doctors to train and refresh decision-making and teamwork skills [40,41]. Nevertheless, some of these serious game applications have been designed for laypersons (Table 1) [35,39]. Several studies report these tools to be superior to traditional teaching methods. For instance, a study carried out on nursing students revealed that those who had access to a serious game after a theoretical presentation had better practical performance outcomes [37]. However, it was unclear whether performance improvements were due to exposure to serious games or simply a result of overall increased training time. Another study targeted at advanced life support (ALS) experts focused on the role of serious games as refresher tools (ie, courses were specifically designed for health care providers [HCP] familiar with a particular procedure, but required a review of basic conceptual and practical learning skills to maintain a high proficiency level on that skill) [34,42]. ALS performance was assessed before and 3 months after a serious game session. Results showed that performance at follow-up was significantly higher than performance outcomes at the beginning of the experiment [34]. Hence, serious games may be effective tools to enhance skill retention between practical courses. Another study compared a cardiopulmonary resuscitation (CPR) serious game with a traditional online course and found no difference between the two teaching modalities [38]. These results support the hypothesis that serious games can be valuable tools for first aid training, being at the minimum comparable to traditional methods in terms of outcomes. Furthermore, it is important that these applications are carefully designed in order to be engaging, motivating, and as realistic as possible. In fact, simulation games are preferable to passive instructions only if they provide active commands that motivate the learner’s immersion into content [38,43].

#### Multiplayers

**Figure 2:**
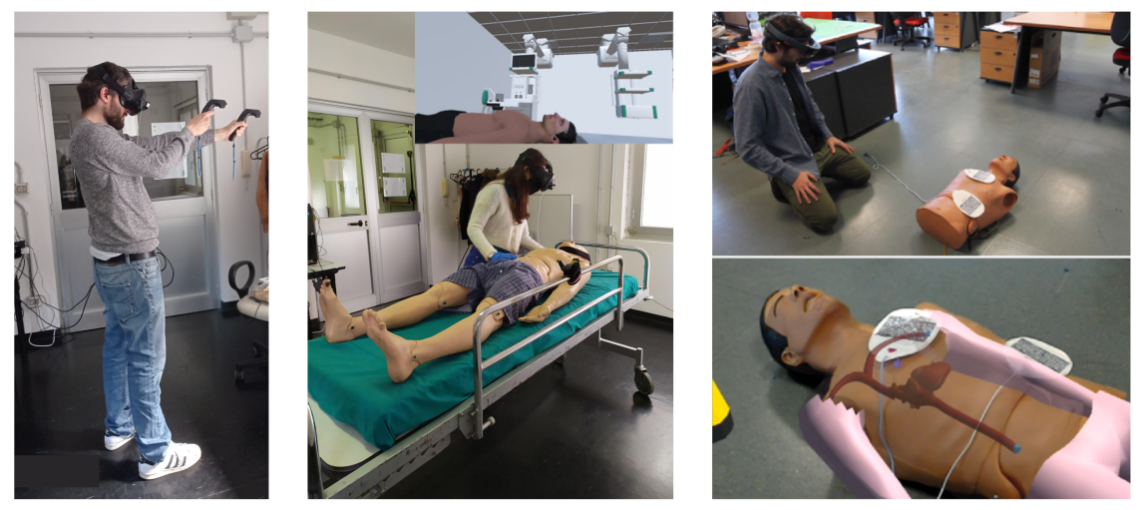
Examples of immersive tools. Left: VR application without haptic feedback. Center: MR system which combines an HMD with a manikin for a more realistic simulation experience. Right: AR application (Holo BLSD) designed to augment a physical manikin with a virtual representation [75]. AR: augmented reality; HMD: head-mounted display; MR: mixed reality; VR: virtual reality.

Serious games involving multiple players are usually called collaborative virtual environments or multiplayer virtual worlds [44,45]. Typically, users are given specific roles within the game (eg, team leader, nurse, etc; Figure 2) with successful outcomes resulting from designed-in collaboration and communication goals.

As for single-player systems, analyses on multiplayer applications yielded results that are limited and difficult to compare. For example, two studies assessing self-efficacy of medical and high-school students using a commercial serious game detected an increase of confidence [46,47]. Unfortunately, the experimental design included both a lecture and serious game practice, thus making it impossible to discriminate whether the self-efficacy increase in procedure confidence was the result of a single training modality. The same research group also explored whether knowledge acquired through multiplayer games would be retained and transferred to manual practice [33]. Briefly, groups of medical students were trained using serious games 6 and 18 months prior to a high-fidelity simulation. Data from these participants were compared to a control group that had not practiced prior to the simulation [33]. Interestingly, all groups showed an improvement in theoretical knowledge. The effect of prior practice became a study factor when the authors took into consideration the number of violations to the CPR guidelines [33], further supporting the idea that serious games are helpful learning tools to learn and refresh CPR algorithm, but not for enhancing the theoretical and practical knowledge related to life-saving skills. Finally, comparing team-leadership skills following either virtual practice or high-fidelity simulation resulted in similar improvements [32,48].

One of the biggest limitations of serious games is the lack of physical practice, as the interaction is limited to keyboard and mouse clicks. To overcome this constraint, a research group added a haptic device (providing physical feedback) to simulate chest compression, thus allowing for a more realistic simulation and improved manual skills [36,45,49]. Yet, since multiple users are assigned different roles, some did not have access to haptic feedback; this resulted in different simulation experiences among trainees. In fact, multiplayer serious games raise an important question: how can students who are practicing different tasks retain the same skills? In other words, it is important to study whether skills acquisition is similar when learners are actively involved in the task, as well as during observational sessions of their practicing peers. On the one hand, team training may be beneficial, as it makes the learning experience more engaging and unpredictable. This is also supported by a qualitative study assessing medical students’ experiences during serious game practice where learners found team simulation challenging, competitive and rewarding, thus leading to better retention [44]. On the other hand, students might be easily distracted when not directly involved in the case presented in the simulation. A study comparing the outcome of students playing different roles during a high-fidelity simulation reported that operative roles enhanced problem-solving, support, and guided reflection abilities [50]. Also, the learning attitude seems related to the role played during the simulation [50]. Further, it is important that medical students learn how successfully be part of a team, as health care is currently provided by multidisciplinary teams who need to work together, and lack of communication and poor teamwork have been related to poor medical care [51]. Within this framework, simulation roles should be rotated, assigning students different roles, so that by the end of the practice session, all participants had experienced the same situations, learning either different skills or the same one from different perspectives. However, in order for the training to be effective, it should not be boring [9-11]. Indeed, one of the main advantages of serious games, and more generally of VR, is the unpredictability embedded within the simulation, thus compelling an increased level of focus and attention on the part of the learner, resulting in better outcomes. Given the foregoing, it appears to be a need for additional studies assessing whether serious games are superior to traditional teaching methods after the “wow” effect has subsided in order to accurately determine the real learning improvement potential of these tools.

#### Semi-Immersive Experiences

The first example of a semi-immersive VR tool was JUST VR, a semi-immersive tool used for the training of nonprofessionals [3]. In this simulation, users face a screen that presents an interactive medical emergency. During the simulation, trainees can move; this is enabled by a magnetic sensor placed on their head; also, they can vocally interact with a virtual assistant (controlled by a technician) who is giving commands on how to manage the emergency situation [3]. Even though this proof of concept might be helpful for learning within the CPR algorithm, it does not provide haptic feedback or effectively train for manual skills.

A recent study presented a CPR training simulation that occurred in the Octave VR facility at the University of Salford, Manchester, United Kingdom [52]. The Octave VR facility is an evolved version of a VR Cave, which is a room equipped with either projectors or screens covering three to six walls of a room. The Octave is defined by an octagonal space that displays an outdoor environment projected onto the walls and floor of the room; students can view a functional CPR manikin at the room’s center and, through shutter glasses, they experience 3D visual cues [52]. Confidence levels in performing lifesaving tasks and performance of second-year nursing students were compared in three environments: the Octave, a skill room (ie, a simulated hospital room), and a simulation room equipped with projectors displaying realistic images and audio of an outdoor urban environment [52]. Surprisingly, self-confidence was lower when students experienced the Octave with respect to the other environments, although students' performance outcomes improved. One possible explanation of these results is that students were unfamiliar with the Octave technology and thus felt less confident than they actually were due to the novelty of the simulation. Indeed, the Octave challenged the trainees more than other simulation technologies, proving to be an effective way to prepare learners for real-life scenarios [52]. However, the Octave technology is expensive and requires dedicated equipment, space, and trained technicians to operate, making it impractical for medical simulation centers to deploy for the immediate future.

A very different application of VR for CPR training is CPRBuddy proposed by [53]; it consists of a virtual avatar displayed on a screen. CPRBuddy follows the users’ performance on a manikin, providing real-time audio and gestural feedback. Interestingly, the avatar is able to illustrate an incorrect action committed by the student before showing him/her the correct procedure. CPRBuddy was tested on a sample of nine novice learners who were asked to perform chest compression before and after the training with the system, showing improved performance after the training. Similarly, Tian et al [54] presented a proof of concept of a CPR trainer which used a Microsoft Kinect and a haptic device. The Kinect captured the user’s movements while performing CPR with the haptic device, which provided the realistic physical feedback of a chest compression procedure. A virtual representation of the user reanimating a virtual patient was displayed on a screen; visual and audio cues were also provided [54]. Even if these are preliminary studies, they suggest that a virtual trainer can effectively support live tutors, reducing their effort during CPR courses and increasing learning outcomes.

#### Immersive Applications

The first example of an immersive VR application for first aid training was presented at the IEEE Virtual Reality Annual International Symposium of 1998 [55]. The first prototype called MediSim was developed to train front-line medical personnel in battlefield medicine. The prototype included a VR HMD, four trackers positioned on the user’s body, and a dynamic causality model that provided simulated changes in a patient’s condition according to the learner’s actions [55]. From the position of the trackers, MediSim reconstructed the trainee body configuration and position, creating a virtual avatar that could interact with a virtual patient and other objects (eg, surgical gloves). No tests studies were reported on the effectiveness of simulated patients or student learning performance, as the main goal of this study was to integrate the system into a battlefield simulator. MediSIM eventually evolved into BioSimMER [56], an application used to train medical first responders to an act of bioterrorism. With BioSimMER, trainees learned how to triage and treat different injuries related to a terroristic attack while at the same time protecting themselves from harm.

After these pioneer studies, research on semi-immersive and immersive VR/MR tools for life support training has been sparse, with only two main studies published between 2000 and 2014 [3,57].

VR applications lacking haptic feedback prove to be efficient learning tools for HCPs who need to refresh skills, be informed of new guidelines, and train for leadership or communicative skills. Moreover, VR applications are promising tools for teaching nonexperts, especially younger people, how to manage medical emergencies. In particular, young people are more prone to use mobile and gaming apps for learning new skills, thus making them a good way to reach them and raise awareness on the importance of first aid knowledge among the general population. In fact, as HMDs have become more common, game developers and designers have begun to create games that increase awareness of life-saving skills. Among these toolsets is *Relieve* (Studio Evil), a science fiction adventure game published by the company that developed VR CPR [58], and an MR application in collaboration with the Italian Resuscitation Council. Other examples include *Accident: The Pilot* (Duality SA), *Ambulance Simulator* (Image Power SA), *Reanimation Inc.* (Nuclear Games), and *Lifesaver VR app* endorsed by the UK Resuscitation Council [59].

Despite growing interest in immersive simulators for CPR training, few studies have assessed their potentialities. Table 2 summarizes the main immersive VR and MR tools for first aid training. A German group has recently implemented VReanimate II, an immersive tool for first aid and reanimation training [60,61]. This application provides different modalities, including a tutorial and two levels of exercises covering different scenarios. The application was tested on users who had limited first aid experience, resulting in a 20% increase in CPR knowledge after training; this suggests that VR has a strong potential to increase functional first aid knowledge. In addition, two studies from 2019 compared VR with a video-based and tablet-based serious game application in order to assess whether people retained more knowledge when trained using the VR system [41,62]. Results showed that the VR application led to increased knowledge of CPR steps [41,62] but a worsening outcome in chest compression technique after training [62]. Altogether, literature on VR for first aid training supports the claim that such technology can be a powerful tool that increases public awareness and learning of life-saving skills, primarily due to the high level of immersivity, perceptual access to real-time scenarios, and impersonation [63]. Further, VR appears to be highly valued by both untrained and expert users who can use it in different ways: the former can learn how to react to a medical emergency, memorizing the CPR algorithm and thus acquiring communicative skills; the latter can easily and rapidly refresh previously acquired knowledge [60,62,64].

**Table 2:** Virtual reality and mixed reality simulations.

Study	Target	Type	Setup	Skill	Status	Design (# subjects)	Kirkpatrick's level
Blome et al [61], 2017; Bucher et al [20], 2019 *VReanimate*	NP^a^	VR^b^	HTC Vive	Chest compression (qualitative); defibrillation	Tutorial and exercises modes; simple instructions without complex text; qualitative evaluation	Usability (8); prepost (22)	1
Wong et al [64], 2018; *CPR + AED* *VR*	HCP^c^	VR	HTC Vive	CPR^d^ algorithm	Tutorial and simulation modes; training on AED^e^; manual skills are performed by an avatar and not by the user; qualitative evaluation	Usability (30)	2b
Aksoy et al [41], 2019; *3DMedSim VR-based BLS*	HCP	VR	HMD^f^	CPR algorithm	Two modes: training which guides the trainees during the emergency and self-test; quantitative Evaluation	Randomized (40)	2b
Leary et al [62], 2019 *VR mApp*	NP	VR	Smartphone (VR), HMD and manikin (MR)	CPR algorithm; defibrillation	Portable and low-cost; quantitative evaluation	Randomized (103)	2a
Vaughan et al [65], 2019	NP	VR, MR^g^	HMD and manikin (MR) or smartphone (VR)	None	Portable; proof of concept	N/A^h^	N/A
Buttussi et al [66], 2020	NP	VR, MR	HTC Vive and manikin (for MR only)	CPR algorithm; chest compression	Training and evaluation modes; training mode provides progressively decreasing clues; quantitative evaluation	Prepost (30)	2b
Semeraro et al [57], 2009 *VREM*	HCP	MR	Physical manikin, data gloves and HMD	None	Proof of concept	N/A	N/A
Almousa et al [67], 2019	HCP, NP	MR	HTC Vive and physical manikin	Chest compression; defibrillation	Multiple scenarios and increasing difficulty levels; animations controlled by a technician; qualitative evaluation	Usability (20)	N/A
Bench et. al [68], 2019 *Code Blue*	NP	MR	HTC Vive and physical manikin	Chest compression	Qualitative evaluation;quantitative evaluation	Prepost (23)	2a
Girau et al [69], 2019	HCP	MR	HTC Vive, Leap Motion, and physical manikin	None	Interactions between the virtual patient and the trainees; proof of concept	N/A	N/A
Leary et al [70], 2019 *VR SCA*	NP	MR	HTC Vive and physical manikin (MR)	CPR; algorithm	Manual and vocal interaction; quantitative evaluation	Prepost (119)	1
Liyanage et al [71], 2019	HCP	MR	HTC Vive, Leap Motion, and physical manikin	Chest compression	Proof of concept	N/A	N/A
Semeraro et al [58], 2019	HCP, NP	MR	HTC Vive and physical manikin	Chest compression	Quantitative evaluation	N/A	N/A

^a^NP: nonprofessionals.

^b^VR: virtual reality.

^c^HCP: health care providers.

^d^CPR: cardiopulmonary resuscitation.

^e^AED: automated external defibrillator.

^f^HMD: head-mounted display.

^g^MR: mixed reality.

^h^N/A: not applicable.

#### Mixed Reality Devices

Currently, literature on virtual reality for life support training is focused on MR systems for CPR training. In other words, many research groups have been trying to “enhance” VR by combining physical elements with a virtual environment. This way, the user interaction is more realistic than by using VR alone. On the one hand, MR tools may be more desirable for life support training, given their inherent advantage of haptic feedback combined with VR; on the other hand, a recent study comparing VR and MR CPR training reported no differences relative to procedural knowledge and self-efficacy [37].

In general, MR tools (Table 2) provide realistic feedback into an immersive environment (Figure 2), being more desirable than AR. By definition, AR integrates virtual and real elements in the learner’s field of view, presenting these elements as two separate layers [72]; However, in order for a technology to be truly immersive, the human brain should not be able to determine the difference between virtual and real elements [73,74]. In this context, MR seems more appropriate since the brain can perceive the environment as it would normally do in the real world, given that all the elements of the scene are virtual [73]; in addition, users perceive haptic feedback and perform actions in a realistic way.

The first example of an MR tool for CPR training is the virtual reality enhanced mannequin (VREM), which combines a half-body manikin with HMD, data gloves, and tracking devices [57]. During the simulation, the user is immersed in a VR environment, but at the same time, they can physically interact with the manikin, as his/her hands’ movements are tracked in real time [57]. Despite having some limitations, such as the lack of performance evaluation, VREM was the first example of MR that combined immersive VR with manikins traditionally used to teach lifesaving skills. VREM was the sole example of an MR simulator until 2018 when new developments and tools appeared. In particular, several research groups implemented MR prototypes that share the same concept of augmenting a VR application with a manikin (Table 2). All these examples combine a manikin, either half-body or full-body, with an HMD device (typically HTC Vive) and a VR application developed in Unity game engine. Despite some differences among the projects, the main idea is to overlap a manikin with its virtual representation in ways that enable trainees to be immersed in a realistic virtual environment accompanied by realistic haptic feedback (Figure 2). The majority of these tools use trackers provided by the VR setup to monitor chest compressions by virtue of their direct placement into the physical hands and/or on the wrists of learners [58,66-68]. To prevent obstruction of the user’s hands, two setups include a Leap Motion device, specifically designed to track the hands in real time [69,71,76]. Leap Motion is attached to the HMD and combines infrared cameras with light-emitting diodes. A proprietary software analyzes the frames captured to extract hand-movement information [76]. These projects are proofs of concept which do not monitor CPR performance but address different challenges of MR, such as the interaction between the user and the manikin [69] and the monitoring of user’s performance via Leap Motion data [71]. Another project [65] attempted to import an MR application designed to be used with an HMD into a Google Cardboard VR platform in order to demonstrate that MR can be used with a smartphone, thus making it even more accessible.

MR solutions highlight an important point that there is a lack of integration between VR and CPR manikin equipped with sensors. In other words, the projects aim at combining VR with physical manikins into a single learning tool, but the two parts remain disconnected, with no data-sharing between them. To overcome this limitation, some research groups used trackers to monitor performance, while others added sensors into the manikin. CPR manikins are largely distributed by a few companies, and this may have caused a lack of communication between hardware and software, suggesting an investigation into how we might better integrate these learning toolsets is necessary. Data recorded by the manikins are protected; therefore, using manikins in combination with VR requires more successful collaborations between research groups and companies. It is unclear whether companies are not interested in providing secure access to their data or, instead, if universities are not willing to share their ideas with commercial, for-profit enterprises. In any case, university and for-profit enterprise collaborations are desirable for various reasons: first, the integration of performance data with VR leads to improved user experiences, as the learner would not be required to wear additional tracking devices; further, the virtual manikin can react more accurately to maneuvers performed by the learner during first aid training. Second, CPR manikins have been used for a long time, meaning that the hardware is stable with standardized performance indexes. The use of these devices would make comparisons with traditional methods more valuable than designing custom-made manikins that require validation prior to being integrated into the VR. Additionally, VR is becoming a common technology within medical simulation environments, as it provides riskless experiences that are controlled and scaled to the user’s ability. Hence, it is likely that companies need to look further into the integration of VR into first aid training scenarios in the near future. Initiating collaborations with universities would speed up this much-needed technological evolution; in particular, commercial and nonprofit collaborations can lead to now medical training opportunities and additional profit for commercial enterprise.

### Augmented Reality

#### Projection-Based Systems

The use of AR within the context of first aid training is a recently recognized challenge. The first AR application to CPR training combined a physical manikin with AR interactive projections [77]. Briefly, sensors located on the manikin were used to monitor chest compression, head position, and airflow; an RGB-D (red, green, blue-depth) camera recorded the user’s position. Finally, a realistic scenario was projected in the simulation room to increase realism and immersivity. This system combined data from the camera and the manikin, giving real-time feedback about the trainee’s performance and position. Similarly, Kwon [78] implemented a portable version of the above prototype where projections are replaced by a mobile phone screen. However, the study did not include any demographic information for study participants; therefore, it is not known whether novice or expert learners can benefit from the AR projections in similar ways.

#### Low-Cost Prototypes

There is increasing awareness that first aid training should be affordable and easy to access, given that a medical emergency can occur anywhere, at any time. Also, CPR training should be tailored to the audience, with high-fidelity, highly functional simulations designed for HCPs in simulation centers and low-cost solutions devoted to the general population or to guarantee optimal training in low-income countries and rural areas. Within the foregoing contexts, some recent proofs of concept have appeared even though validation studies assessing the efficacy of such ideas are still missing. In 2016, Philips presented a low-cost project for automated external defibrillator placement training [79]. The system included a cloth sheet representation of a patient and a camera that monitored the correct positioning of the electrode pads. Feedback on the pads’ location and hand position were shown on a monitor. The rationale behind the project was that an effective CPR response in untrained personnel should be automatic and related to muscle memory rather than abstract cognitive learning. In this model, resistance that mimics chest stiffness should be included, in addition to hand and pad positioning. A more recent study presented the first prototype of an AR application usable with a smartphone [80]. The system combined two markers with a pillow mimicking the chest via a mobile application and a smartphone. The application computes chest compression depth and rate using markers and projects information via smartphone to a body over the pillow, blending the digital body into the real world. Altogether, studies showing the potentialities of low-cost AR applications suggest that further validation and efficacy studies are required prior to using these systems for CPR training. As an example, [80] have neither tested the system with experts nor compared their system with a traditional manikin. Hence, it is difficult to foresee the near-term role that these applications may play in increasing CPR knowledge among the general population.

#### Optical See-Through Applications

In recent years the majority of research studies have focused on AR in emergency training designed applications, taking advantage of optical see-through AR devices like the Microsoft HoloLens and Google Glass (Figure 2) [75,81-86]. AR-based tools can be divided into (1) applications that assist a user who needs to perform a life-saving task [81,85] and (2) applications that augment the simulation experience with virtual elements, giving real-time performance feedback during training [75,82,83]. The first group of tools aims at shortening the rescue time through cues appearing in an emergency setting. So far, in the latter context, experimental study results have been controversial. Siebert [81] compared the time required to provide defibrillation in two groups of pediatric residents. The first group could follow the pediatric advanced life support algorithm on a pocket reference card; the second group used Google Glasses to access the same information. Results show a similar shocking time in the two groups, but a better defibrillation dose delivered by residents using Google Glasses. Conversely, a study carried out on untrained subjects revealed that participants wearing HoloLens reacted faster and better than those having access to an instruction checklist on a tablet [85]. One possible explanation for these results is that AR may be beneficial for novice learners who do not have any previous experience with algorithms and instructions; however, HCPs may get distracted by information appearing in their field of view because they may look for confirmation of their prior knowledge [87]. Another variable may be that health professionals are familiar with the checklists. All of this suggests that AR could be a valuable tool for novice first aid rescuers and could be included in first aid kits. Indeed, further studies are required to (1) assess the acceptance rate of AR among the general population, (2) define its benefits and limitations, and (3) determine whether the efficient use of optical see-through systems to perform lifesaving tasks requires training.

Another class of AR-based applications includes CPR trainers that provide real-time feedback on users’ performance during simulations by combining a real manikin with AR optical see-through devices. Recently, two research groups have implemented similar tools: HoloBLSD [75,86] and CPReality [82,84]. HoloBLSD is a self-instruction basic life support defibrillation trainer having three modalities: learning, rehearsal, and evaluation, allowing the user to practice prior to evaluation [75,86]. CPReality is intended for hands-only CPR training; briefly, information on chest compression is measured by the manikin and integrated into the AR application, which displays, in real time, how the blood flows into the circulatory system as a result of the compression [82]. Both systems have been recently tested among HCPs, comparing the performance of participants who use AR with the performance of subjects undergoing traditional manikin-based training [70,86]. Results show that both HoloBSLD and CPReality lead to performance outcomes similar to those obtained with traditional training methods [84,86]. From a theoretical standpoint, self-learning AR tools can be at the minimum comparable to traditional training methods. However, one important factor should be taken into account: the biggest difference between instructor-guided courses and self-learning is the customization of the courses based on the learner's difficulties and abilities (ie, individualized learning). In particular, during a traditional course, an instructor can adjust the content of a simulation according to audience needs. This means tailoring courses to the audience’s skills, needs, and background. For this reason, it is important to further investigate the role of personalized training as to its impact on learning and retention outcomes. At this time, no current available AR or VR tools provide personalized and self-adjustable programs, as opposed to instructor-led training.

## General Discussion

This review has summarized the main scientific studies related to first aid training using VR and AR. Some of the prototypes investigated have been designed by medical groups, others by technicians. Some of the prototypes focus on leadership and teamwork training, and others have been implemented to enhance the practice of manual skills.

The analysis of the existing AR- and VR-based systems has helped us to identify the primary features that a simulator should have in order to increase individualized outcomes. These studies highlight the importance of choosing the end user prior to defining the main features of a training toolset. In fact, nonprofessionals have little to no medical background, whereas medical students and HCPs require advanced training. Also, the general public is trained at far less frequent rates than medical caregivers. The general public needs more and better information—both theoretical and practical—in learning how to deal with stressful medical emergency situations without the immediate aid of a medical professional. Advanced life support instead requires coordination and cooperation, with everyone involved performing as optimally as possible (Textbox 2).

**Main features an augmented reality or virtual reality-based simulator should have according to its users and purpose.**

**Nonprofessional:**

- Procedures:
  Environmental safety
  Chain of survival activation
  Cardiopulmonary resuscitation performance
  Automatic medical instrument management
- Training:
  Knowledge of procedures
  Stress management
  Manual skills
- Assessment:
  Guarantee safety
  Procedure correct execution
  Manual skills.

**Health care providers:**

- Procedures
  Environmental safety
  Basic life support defibrillator
  Advanced life support
  Manual instrument management
  Diagnostic skills in emergency
  Invasive procedures performance
  Drugs administration
- Training:
  Crisis resource management
  Stress management
  Familiarity with instruments
  Procedure knowledge
  Manual skills
  Diagnostic skills
  Drugs management
  Teamwork
- Assessment:
  Guarantee safety
  Procedure correct execution
  Ability in performing invasive procedures
  Diagnostic performance
  Decision making
  Drugs effects and indication knowledge

Aside from defining the end user, it is important to establish whether the simulation tool is intended for training, assessment, or both, as training and evaluation tools should have different features. For instance, a training tool used by nonmedical learners should include information about how to communicate with paramedics or where to place one’s hands to optimally perform chest compression (Textbox 2). An evaluation instrument should simulate an emergency occurring in a nonhospital setting, also including distractors. In addition, chest compression should be assessed in a quantitative way, measuring compression rate, depth, and recoil. A simulator for HCPs should include pathology and pharmacology notions, as well as diagnostic skills used in emergency and invasive procedures performance (Textbox 2).

At the present time, we are unable to find a clear distinction between nonmedical and training and assessment tools. We believe this is a crucial point in order to effectively design AR/VR-based simulators for life support training. In fact, instructors should tailor the content they are delivering to their audience, in the same way first aid tools should be adapted to the end users, in order to guarantee engaging and challenging training.

A distinctive feature of simulation toolsets is the ability to train small groups of medical students, either in person or via distance learning; this is important for three reasons. First, every participant can take a specific, assigned role during the training. Presently, several multiplayer serious games have been implemented and introduced as medical training tools [32,44,45,48]; further, few multiplayer VR platforms with multiple cases designed for HCPs are available (eg, *SimX*, *OMS Interprofessional*, and *ORama VR*). Still, multiplayer AR and MR tools have not yet been designed. Second, the debriefing phase following the simulation training is more effective if the clinical case study is analyzed from multiple perspectives [88-92]. The majority of the studies we analyzed did not consider the possibility of providing the debriefing phase via the simulator itself, other than giving performance scores or video recordings of the simulation. We believe this would be a feature that would innovate medical simulation as an optimal training strongly affected by an organized debriefing phase that takes into consideration learner’s performance, errors, divergent behaviors, and user experience. Thirdly, at the time of writing, the way medical education is delivered has strongly changed due to the COVID-19 pandemic. Changes include the need to train smaller groups of students, thus replicating the same clinical case scenarios multiple times, and the need to deliver content online, taking advantage of distance learning but maintaining the same level of content quality and engagement. Another point to consider is the importance of designing AR- and VR-based simulators providing haptic feedback and monitoring the user’s manual skills. Among the basic tasks a user needs to perform during first aid, there is chest compression and bag-mask ventilation, in addition to more complex task knowledge required by HCPs (eg, invasive procedures, drugs administration, etc). At present, performance monitoring is sparse and typically limited to hands-only CPR [53,54,57,67,75]. Indeed, research studies in the field should focus more on realistic feedback and performance monitoring systems, considering all the maneuvers associated with optimal first aid.

Importantly, it is also necessary to carry out detailed learning outcome studies aimed at assessing whether AR- and VR-based systems are comparable to traditional training methods. In fact, if VR is equivalent to traditional methods, medical simulation can benefit from VR in terms of setting space, flexibility, and training time. This review indicates that many research studies report proof of concepts without comparing them to the most popular in-use manikin-based simulators [93,94]; specifically, very few studies compared VR/AR tools with traditional training methods, reporting controversial or inconsistent results.

## Conclusions

Research on VR and AR for basic and advanced life support training is heterogeneous in terms of users, type of technology, experimental design, and metrics. Analysis of the existing studies revealed the importance of defining the end user and the purpose of the simulator during the design phase, as professionals and nonprofessionals need to learn different skills. Also, this review highlights a few limitations of the current devices that should be addressed to improve the learning outcome. These include the possibility to develop multiplayer tools, the inclusion of the debriefing phase within the simulation, the ability to monitor users’ performance, and the possibility to provide realistic haptic feedback. In this context, standardized tests are required to assess the benefits of new technologies in life support and, more generally, in medical training.

## Appendix

Multimedia Appendix 1 Supplementary materials.

## Abbreviations

ALS: advanced life support
AR: augmented reality
CPR: cardiopulmonary resuscitation
HCP: health care provider
HMD: head-mounted display
MR: mixed reality
VR: virtual reality
VREM: virtual reality enhanced mannequin
