# Supplementary material for: Viewpoint: Virtual and Augmented Reality in Basic and Advanced Life Support Training
Source: JMIR Serious Games. 2022 Mar 23;10(1):e28595. doi: 10.2196/28595 (PMC8987970; doi:10.2196/28595)
Supplement: Multimedia Appendix 1 [file games_v10i1e28595_app1.doc]

## SUPPLEMENTARY MATERIALS

## Inclusion Criteria

The goal of this work is to provide a comprehensive analysis and overview of state-of-the-art of VR- and AR-based simulators for life support training. We also aimed at describing different technologies and trying to standardize the language of studies coming from multiple research areas, such as medicine, engineering and computer science. We included articles published either in journals or conferences and written in English. Our research was carried out on PubMed and Google Scholar. The former mainly includes article written by physicians, medical instructors or simulation specialists; the latter also includes works of engineers and computer scientists. As our goal was to find articles describing AR- and VR-based simulators for life support training, we used the following keywords: AR, VR, first aid, CPR, ALS, BLS, BLSD, sudden cardiac arrest, life support training. We selected 16 articles (10 different applications described) on serious games (12 journal articles, 4 conference papers); 18 articles on 17 semi-immersive and immersive VR (12 journal papers and 6 conference articles), and 11 articles describing 8 different AR applications for life support training (6 journal articles, 5 conferences articles). Also, we searched online for VR applications on life support training developed by game designers, and we selected 5 applications to raise awareness on life support training.

Supplementary Table 1. Semi-immersive VR and AR systems. The first column indicates the study and the name of the application, if any. The second column indicates the target the simulator is designed for (HP: healthcare providers; NP: nonprofessionals). Column 3 indicates the type of the of the simulator (S-VR: semi-immersive VR; PB-AR: projection-based AR; LC-AR: low-cost AR; OST-AR: optical see-through AR). Columns 4, and 5 specify the setup and the skills trained. Column 6 indicates whether the study is a proof of concept or if it is qualitatively or quantitatively tested. Colum 7 shows the study design and the number of participants tested; the last column reports the evaluation of the outcome, according to Kirkpatrick model. Level 1: satisfaction; Level 2a: acquisition of self-confidence; level 2b: acquisition of knowledge and skills; level 2c: retention of knowledge and skills over a period of time; level 3: behavioral change, level 4: patient outcomes.

| **Study** | **Target** | **Type** | **Setup** | **Skill** | **Design** | **Kirkpatrick**  **Level** |
| --- | --- | --- | --- | --- | --- | --- |
|  |  |  |  |  |  |  |
| Ponder et al. 2002  *JUST VR* | NP | S-VR | Screen. Magnetic sensor placed on the user’s head allowing free movements.  Vocal interaction with a virtual assistant controlled by a technician | Decision training CPR algorithm | Proof of concept | N/A |
| Rushton at al.  *2020* | HP | S-VR | Octave: octagonal space. outdoor environment projected onto the walls and floor and, through shutter glasses they experienced 3D visual cues | BLS | Mixed-method (208) | 3 |
| Morrison-Smith et al. 2018  *CPRBuddy* | NP | S-VR | Virtual avatar displayed on a screen following the users’ performance on a manikin, providing real time audio and gestural feedback | Chest compressions | Pre-post (9) | 1 |
| Tian et al.  2014 | NP | S-VR | Microsoft Kinect capturing user’s movement, screen and haptic device | Chest  compression | Proof of  concept | N/A |
| Park et al. 2013 | NP | PB-AR | Physical manikin with AR interactive projections. Sensors on the manikin monitor chest compression, head position and air flow; an RGB-D camera recorded the user’s position | CPR | Proof of  concept | N/A |
| Kwon et al.  2014  *HeartiSense* | NP,  HP | PB-AR | Physical manikin with AR visible via mobile phone screen Visual and audio feedback and post training evaluation | CPR | Mixed-method (25) | 2c |
| Djajadiningrat  et al. 2016  *Virtual trainer* | NP | LC-AR | Cloth sheet representation of a patient and a camera monitoring the positioning of the electrode pads. Feedback shown on a monitor | AED positioning | Proof of concept | N/A |
| Boonbrahm et  al. 2019 | NP | LC-AR | Two markers, a pillow mimicking the chest and a mobile application computing chest compression | Chest compression | Proof of concept | N/A |
| Siebert et al.  2017 | HP | OST-AR | AR glasses showing PALS algorithm steps | PALS | Randomized (20) | 1 |
| Johnson et al.  2018  *HoloCPR* | NP | OST-AR | Instruction on how to perform CPR and position AED are shown via Microsoft Hololens | CPR, AED | Mixed-method (42) | 2a |
| Strada 2019,  Ingrassia et al.  2020  *Holo-BLSD* | HP | OST-AR | Real manikin, AR which  provides performance feedback | BLSD | Usability (26) | 1 |
| Balian et al.  2019; Leary et  al. 2019;  Leary et al.  2020  *CPReality* | HP | OST-AR | Information on chest compression is integrated into the AR application which displays, in real time, how the blood flows | Chest compression | Mixed-model (100) | 2a |
